# Diseases Admitted as in and Out-Patients of the Physicians of the Westminster Hospital, between the 20th of April and the 20th of May

**Published:** 1799-06

**Authors:** 


					General Remarks cn the prevailing Difeafes of London. 335
Difcajb: admitted as in and out-Patients of the Phyficians of the Wejl-
minjier Hofpital, between the 10th of April and the lotn of May %
No. of Cases.
T yphus - - ix
Tertian - - w I
Quotidian - i
Scarlatina - 1
Amenorrhea - - 1
Afcites 1
Afthenia - - 5
Afthma -_ - ? - ? 2
Catarrh - - 1
Colic - -? I
Convulnons - 1
Cough - - 9
Diarrhoea 4.
DyTpepfia - - - 4
Enterodynia - - 2
Epifiaxis - _ - - 1
No. of Cases.
Eryfipeks - I
Hooping-cough - ' - 1
Hremoptoe - 2
Hyfteria 2
Itch - - "4
Lichen - i
Lumbago 1
Leucorrhcea - 2
Menorrhagia 1
Phthifis 3
Pleurify I
P.heumatifm - 14.
Struma - - 3
Tinea - - 2
"Urticaria - - 7-

				

